# A Dialogic Participatory Model Between Professionals and Patients for the Co‐Creation of Transitioning Care Management Programmes in Rare Bone Diseases

**DOI:** 10.1111/hex.70758

**Published:** 2026-07-14

**Authors:** Marina Mordenti, Silvia Forni, Eleonora Grippa, Maria Cecilia la Forgia, Davide Scognamiglio, Manila Boarini, Gabriella Massa, Andrea Romeo, Alessandro Sergi, Luca Sangiorgi, Transitioning Care Management Working Group (TCM WG), Giovanni Beltrami, Domenico Campanacci, Nunzio Catena, Mauro Celli, Marta Cervo, Leonardo D'Agruma, Gian Luigi Federico, Giovanni Gallone, Michela Veronika Gonfiantini, Laura Masi, Maria Beatrice Michelis, Osvaldo Palmacci, Annabruna Ronchetti, Paolo Trezza

**Affiliations:** ^1^ Department of Rare Skeletal Disorders IRCCS Istituto Ortopedico Rizzoli Bologna Italy; ^2^ Associazione Conto alla Rovescia, ACAR Aps Roma Italy; ^3^ Alma Mater Studiorum Università di Bologna Bologna Italy; ^4^ Azienda Usl Toscana Nord‐Ovest Pisa Italy; ^5^ Università di Firenze, AOU Meyer Firenze Italy; ^6^ Università di Firenze, AOU Careggi Firenze Italy; ^7^ IRCCS Istituto Giannina Gaslini Genova Italy; ^8^ Policlinico Umberto Roma Italy; ^9^ AOU Meyer Firenze Italy; ^10^ Fondazione IRCCS “Casa Sollievo della Sofferenza” San Giovanni Rotondo Italy; ^11^ AORN Santobono Napoli Italy; ^12^ IRCCS Istituto Ortopedico Rizzoli Bologna Italy; ^13^ Ospedale Pediatrico Bambino Gesù Roma Italy; ^14^ Fondazione Policlinico Universitario A. Gemelli IRCCS, Università Cattolica del Sacro Cuore Roma Italy; ^15^ Presidio Ospedaliero Gaetano Pini Milano Italy

**Keywords:** co‐creative approach, Maffucci syndrome, multiple osteochondromas, ollier disease, operational solutions, rare bone diseases, transitioning care management

## Abstract

**Introduction:**

Rare diseases are a group of heterogeneous conditions affecting fewer than 5 per 10,000 individuals in Europe, with rare bone diseases representing a clinically significant subgroup. Multiple osteochondromas, Ollier disease and Maffucci syndrome are multifocal benign rare disorders, characterised by bone deformities, functional limitations, with symptoms arising early in life and chronically progressing. Proper transition planned programmes to accompany patients moving from childhood to adulthood are limited. This study aims to describe the steps taken to establish a shared consensus on recommended actions for transitioning, by implementing a co‐creative approach that involves healthcare professionals, a patient organisation (ACAR Aps), patients and families.

**Methods:**

The first step was the definition and development of a dialogic participatory model (DPM) performed by ACAR Aps with the guidance of an expert in healthcare management, which supported the definition of a guiding question. The second step was composed of a set of multidisciplinary brainstorming sessions aiming at answering the guiding question. The ACAR Aps were responsible for the third step, which consisted of the organisation and summary of the brainstorming sessions, leading to preliminary operational solutions. The final step comprised the collective validation during the patient organisation meeting. This discussion involved experts and members of the ACAR Aps community, providing an open forum to share, discuss and refine the preliminary recommendations.

**Results:**

The DPM resulted in the definition of 11 operational solutions to improve transitional care for patients with MO, OD and MS organised according to the entity primarily responsible for their implementation. These solutions constitute measures to address patients’ priorities in the short‐ and medium‐term.

**Conclusion:**

The entire process represents a structured yet flexible environment for collaborative consensus‐building and for the establishment of actionable, achievable and community‐endorsed solutions.

**Patient or Public Contribution:**

This study was conceived, designed and conducted by the ACAR Aps patient organisation. Patients and caregivers played a pivotal role in the research process, actively participating in roundtable discussions during the association's national convention. Their insights and lived experiences were instrumental in reviewing and refining the study's contents, ensuring that the findings accurately reflect the priorities and perspectives of the community.

## 1 Introduction

Rare diseases (RDs) are a group of heterogeneous conditions affecting fewer than 5 per 10,000 individuals in the European Union [1] and fewer than 200,000 people in the United States [2]. Rare bone diseases (RBDs) represent a clinically significant subgroup, often leading to severe skeletal deformities, impaired mobility and reduced quality of life. Due to their low prevalence, patients with RDs face considerable challenges in terms of timely diagnosis, clinical management and therapeutic development [3]. Consequently, they represent a critical area of unmet medical needs within global healthcare systems.

Multiple Osteochondromas (MO—MIM #133700, #133701), Ollier Disease (OD—MIM #166000), and Maffucci syndrome (MS—MIM #614569) are multifocal benign disorders that usually appear in childhood and frequently cause bone deformities and functional limitations. MO is a rare autosomal dominant disorder whose key manifestation is the presence of bony cartilage‐capped protrusions [4, 5]. OD and MS are two ultra‐rare forms of enchondromatosis with an estimated prevalence of one in 100,000. The presence of several benign growths, called enchondromas, is the key sign of these conditions. In MS, enchondromas are found with vascular anomalies, including cutaneous, soft tissue or visceral haemangiomas. Up to 50% of patients with either OD or MS experience malignant transformation [4, 6].

For MO, OD and MS, as well as for several RBDs, the disease onset often occurs early in life and progresses chronically throughout adulthood. While there are notable differences depending on the specific RBD, many patients experience symptoms during childhood that persist and evolve throughout their lives, impacting their physical health, psychology and personal lives. Furthermore, the transition to adulthood may give rise to new requirements, necessitating the consultation of dedicated specialists in areas such as reproduction, malignant transformation and metabolic issues [7].

Despite the existence of transition programmes to support and accompany patients moving from childhood to adulthood, they cover only a limited number of conditions and few adolescents—and their relatives—receive support during this complex process [8, 9, 10].

In 2024, Scognamiglio et al. presented an open dialogue approach (ODA) to explore the priorities for patients, relatives, caregivers and healthcare professionals (HCPs) [11]. This study highlighted three main priorities—psychological support, defined care plan/pathway and accessible digital health record—all aspects of the pivotal process of transitioning care management (TCM) from childhood to adulthood for MO, OD, MS patients and families. The absence of specific guidelines and recommendations for these diseases and, in general, for many bone diseases was the starting point for this study. Despite the presence of certain limitations, the study demonstrated the benefits of convening all stakeholders together within an ODA to identify shared priorities and approaches, and to propose a list of key issues during the delicate process of transition.

The conceptualisation, promotion and execution of this ODA were carried out by an Italian patient organisation (PO) named ‘Associazione Conto Alla Rovescia’ (ACAR Aps), which is devoted to multiple osteochondromas and enchondromatoses. The ACAR Aps [12] was founded in 2006 with the aim to serve as a bridge between patients and families, HCPs, researchers and institutions, promoting the dissemination and sharing of knowledge. ACAR Aps is entirely composed of, led by, and operated by patients with MO, OD or MS and their family members, all acting on a voluntary basis. As such, the organisation itself constitutes an expression of lived experience, consistent with the principles of community‐based participatory research (CBPR), in which the community acts as co‐investigator rather than as a passive subject of enquiry [11]. Building on these foundations and taking into consideration the emerging needs and priorities, ACAR Aps has proceeded with the implementation of its activities based on the ODA results [12]. The objective of this study was to describe the subsequent steps performed in establishing a shared consensus in terms of recommended actions for TCM, by implementing a co‐creative approach that involved patients and families, as well as HCPs and researchers from reference centres for MO, OD and MS.

## 2 Methods

**Figure 1:**
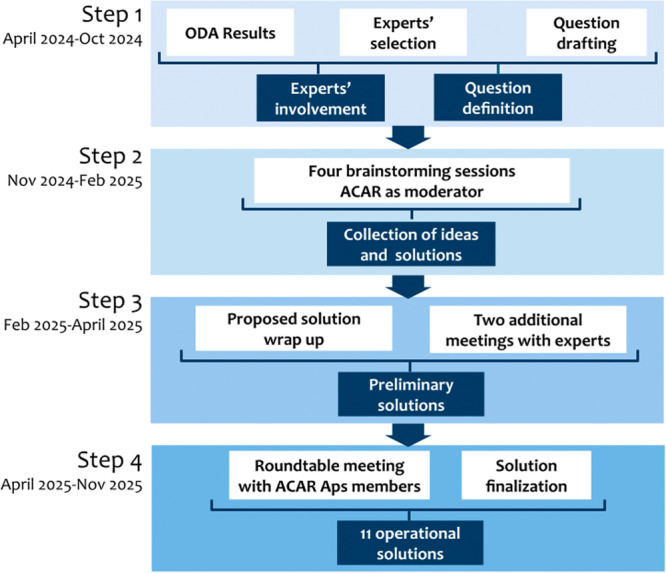
Flowchart of project steps: For each step, actions (white boxes) and outputs (blue boxes) are reported.

Building on the ODA results [12], the subsequent ACAR Aps initiative aimed to translate the priorities that emerged into operational solutions and to improve transitional care for patients with MO, OD and MS. The process was structured as a sequence of interdependent steps, with each step informed by the outcomes of the preceding one. ACAR Aps coordinated the overall process, facilitating interactions among professionals and ensuring that governance activities incorporated both clinical expertise and patient‐reported experience (Figure 1).

### 2.1 Step 1: Conceptualisation and Set Up of the Dialogic Participatory Model (DPM)

A DPM was defined and developed by ACAR Aps with the guidance of a medical doctor expert in healthcare management (AS), in order to accomplish three core principles. The first principle was the feasibility of operational solutions in real‐world settings, achievable within a short timeframe and characterised by low economic and organisational impact. The second principle was the achievement of a shared consensus among multiple stakeholders on TCM, involving experts from different disciplines, ensuring a multidimensional perspective and the identification of feasible actions. The third and final principle was maintaining an integrated co‐creative approach and network‐based principles to synthesise heterogeneous perspectives and experiential knowledge, while promoting inclusivity, transparency and shared responsibility among stakeholders.

The DPM was conceived in alignment with two established participatory research frameworks. Firstly, it draws on the principles of CBPR, which foregrounds equitable partnership between the community and other stakeholders, recognising community members as co‐investigators who contribute their experiential knowledge to all aspects of the research process [11]. Secondly, it draws on the co‐production framework as conceptualised in healthcare contexts, which positions patients and HCPs as joint producers of knowledge and solutions rather than as service recipients and providers operating in separate domains [13]. These frameworks were selected as they most closely reflect the organisational nature of ACAR Aps and the iterative, multi‐stakeholder structure of the process described herein. Taking into consideration those principles, during the set‐up phase of the DPM, a multidisciplinary panel of experts in MO, OD and MS was invited to participate in structured brainstorming sessions. The guiding question for the brainstorming meetings was defined during an in‐depth discussion between ACAR Aps and the expert in healthcare management (AS):

In your work setting, which activities or strategies could be implemented in the coming months to ensure an effective transition of patients from paediatric to adult care, with a focus on concrete and feasible proposals?.

The panel was initially formed by inviting 16 HCPs and researchers from nine reference centres selected on the basis of previous collaborations and their demonstrated interest in participating. In addition, these panellists were encouraged to extend the invitation to other specialists in order to involve different disciplines related to the conditions considered. The final panel consisted of 11 experts from various disciplines—including 5 orthopaedic surgeons, 1 endocrinologist, 1 physiatrist, 1 psychologist, 1 biologist and 2 paediatricians—affiliated with 7 specialised centres across Italy (Table 1). Of the 16 initially invited professionals, 11 confirmed participation. The five non‐respondents did not provide reasons for their non‐participation, making it not possible to formally exclude the presence of a response bias.

**Table 1:** Project plan description: Steps and participant.

	STEP 1: Conceptualisation and set up	STEP 2‐3: Brainstorming sessions, wrap‐up and internal validation	STEP 4: Preliminary dissemination and collective validation	TMC working group
ACAR Aps	Board and scientific committee (SF, EG project leaders); AS (expert in healthcare management)	SF, EG, AR	SF (moderator), ACAR community (ca. 150 participants at the Annual Meeting)	SF, EG, AR, MClF, GM, AS
IRCCS Istituto Ortopedico Rizzoli (Bologna)	LS, MM, MB	MM, MB	LS (moderator), MM, MB	LS, MM, MB, GG
Azienda Ospedaliero‐Universitaria Careggi (Firenze)		DAC, LM	EN	DAC, LM
IRCCS Istituto Giannina Gaslini (Genova)		MBM, AR		MBM, AR, NC
Policlinico Umberto I (Roma)		MC		MC
Policlinico Universitario Agostino Gemelli (Roma)		OP	OP	OP
Azienda Socio Sanitaria Territoriale Gaetano Pini ‐ CTO (Milano)		PT		PT
Ospedale Pediatrico Bambino Gesù (Roma)		MG	MG	MG
Azienda Ospedaliero‐Universitaria Meyer (Firenze)				GB, MC
Fondazione IRCCS ‘Casa Sollievo della Sofferenza’ San Giovanni Rotondo (FG)				LdA
AORN Santobono, Napoli				GLF

### 2.2 Step 2: Multidisciplinary Brainstorming Sessions

As mentioned, the operational core of DPM was a participatory technique involving small‐group interaction and iterative peer communication. In the period between November 2024 and February 2025, four online brainstorming sessions were held, with a maximum of four panel professionals participating in each session, with the aim of proposing solutions for the formal question defined in Step 1. ACAR Aps representatives were present to act as moderators and to keep track of all thoughts and interactions. The rules of the brainstorming sessions emphasised respectful dialogue, including avoiding interruptions and refraining from criticism. This approach ensured that all participants were able to express their views and that discussions were conducted in a constructive and open environment.

### 2.3 Step 3: Wrap‐Up and Internal Validation

ACAR Aps was responsible for the organisation and summarisation of the large amount of material collected in Step 2. This objective was accomplished through a series of internal meetings among the organisation's representatives involved in the study.

The analytical process applied to the brainstorming outputs followed an affinity grouping approach: All ideas and proposals generated during the sessions were captured through detailed written notes and session minutes produced by ACAR Aps representatives. These materials were subsequently reviewed during a series of internal meetings, in which thematically related items were progressively clustered into coherent groups. Where divergent perspectives had emerged across sessions, these were documented and addressed through iterative collegial review within ACAR Aps, with convergence sought through discussion and synthesis rather than formal voting. The resulting thematic clusters formed the basis for the preliminary operational solutions brought forward to Step 4.

Two additional rounds of meetings were held in March 2025 with panel experts to enable peer interaction, promote the cross‐validation of ideas collected from the prior sessions and support the identification of feasible, context‐specific strategies for improving TCM. This allowed participating experts to collectively refine several preliminary operational solutions and align them with operational realities across different clinical settings.

### 2.4 Step 4: Preliminary Dissemination and Collective Validation

The results of the previous steps were presented during the ACAR Annual Meeting, which was held between the 11 and 13 April 2025 [13], an event supported also by ERN BOND, the European Reference Network for RBDs [14]. This annual event is devoted to patients and their families, and features scientific presentations on clinical management and updates on research activities by national and international experts in the context of MO, OD and MS. During the meeting, some time is usually dedicated to patients and caregivers to discuss and share experiences and concerns, creating a safe environment for mutual support.

The second day of the meeting concluded with a 2‐h roundtable discussion, during which the identified operational solutions were presented and explored with HCPs, patients and families.

This discussion involved experts and members of the ACAR Aps community, providing an open forum to share, examine and refine the proposed preliminary recommendations. In fact, five experts from the multidisciplinary panel, representing four different institutions, participated in the roundtable, covering five out of six disciplines of interest (see Table 1).

Additionally, a clinician (LS) with extensive experience with MO, OD and MS was identified from the outset and deliberately not involved in Steps 2 and 3. This clinician, together with an ACAR Aps representative (SF), facilitated the roundtable discussion, ensuring that all perspectives and voices were equally considered. The roundtable was recorded, following the collection of specific authorisation for the use of images, with the aim of creating a detailed record of the points discussed and supporting a summary of the identified solutions. All material collected during this phase was subsequently reviewed and finalised with the support of the TMC working group.

This final step represented both a moment of dissemination of the achieved solutions to patients and families and a collective validation step of the DPM, promoting transparency, accountability and alignment among professionals organisation representatives, patients and families.

## 3 Results

As outlined in Steps 2 and 3 of the Methods, the comprehensive process resulted in the identification of preliminary operational solutions for TCM that emerged from the expert panel brainstorming, and were subsequently summarised by ACAR Aps.

Hereafter, the list of the *preliminary solutions,* organised by main topic:

1. The centrality of the young patient and their family is a priority.
  This aspect comprises the gradual accompaniment of the paediatric patient and their family during the transition process, with the aim of reducing the sense of abandonment. This should be complemented by an empowerment strategy for young patients, including education on self‐management and a gradual shift from a passive role to an active one in handling their own health. Furthermore, effective communication between young patients and HCPs is paramount to establishing a stable point of reference. These solutions could also be promoted and endorsed by POs, acknowledging their role as a hub between patients and professionals in different local healthcare settings.
2. Promote a multidisciplinary and intra‐/inter‐hospital collaboration.
  The definition of specific networks between paediatric and adult specialists aimed at facilitating patient management, supported by the scheduling of regular interdepartmental meetings to update professionals on the most complex cases and emerging treatment opportunities. A further key element is the creation of shared outpatient clinics, which facilitates seamless transitions among specialists and coordinated monitoring of patients, particularly for treatments that significantly impact patients, such as surgeries and rehabilitation during the transition to adulthood.
  These preliminary solutions should be implemented both within and between hospitals to ensure a multidisciplinary and holistic approach to care.
3. Develop tools to support the transition between paediatric and adult care.
  This crucial aspect can be supported by actions on several levels. The initial step is to create a Patient Health Profile (PHP), a data‐driven summary that provides a comprehensive view of current conditions and expected challenges of each patient. This PHP should include essential information on disease features to be shared with the patient, their family, and future HCPs, ensuring that the correct information is passed on to the relevant specialists. This document could form part of a broader initiative to develop specific Diagnostic‐Therapeutic Care Pathways (PDTAs) for RDs, involving multiple specialists. Furthermore, the process of identifying and updating lists of referral centres for adults, organised by territory, is integral to ensuring that patients are not left without reference points during the transition.
4. The enhancement of the quality of care is a primary concern.
  The promotion of an integrated approach encompassing clinical aspects, research knowledge and psychological support facilitates a more multidimensional approach to care. This objective can be achieved through the implementation of targeted training programmes for HCPs, focusing on the requirements of patients with RDs and on the promotion of health education strategies that can contribute to enhancing long‐term management. Another key strategy for the promotion of quality of care can be the increased involvement of community‐based healthcare services, which can further support patients and their families locally.
5. A flexible approach to the TCM is recommended.
  Firstly, it is vital to personalise the transition, paying particular attention to age‐based customisation in line with patients’ specific requirements. The creation of more flexible pathways is essential, and these pathways must take into account individual needs, family context and the complexity of the pathology. This aspect can be further promoted by a temporary overlap between paediatric and adult specialists to ensure a more effective transition.

During the patient transition, priority needs can change rapidly, and organisational flexibility is essential to promptly adapt treatment pathways. A case in point is the management of surgical waiting lists, which must adapt to newly emerging needs.

**Figure 2:**
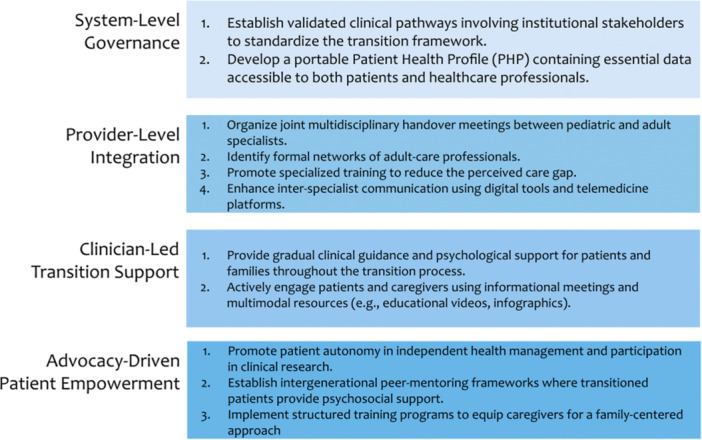
Operational solutions to improve transitional care for patients with MO, OD and MS are grouped by the entity mainly responsible for their implementation.

The preliminary operational solutions for TCM were presented and discussed during the roundtable at the ACAR Annual Meeting, with active involvement from PO members. Subsequently, ACAR Aps produced several videos consisting of highlights from the roundtable, which are available on the organisation's YouTube channel (https://www.acar-aps.org/cosa-facciamo/progetti-e-attivita/ricerca/dialoghi-aperti/) in Italian. Finally, following an extensive review process, ACAR Aps organised and summarised all the aspects discussed into 11 operational solutions (Figure 2), which were clustered into four main groups based on the entity mainly responsible for their implementation.

### 3.1 System‐Level Governance: Policy and Infrastructure

1. Develop and establish a portable and concise Patient Health Profile containing essential patient data—including surgical history, ongoing treatments and clinical contact information—accessible to both patients and HCPs.
2. Develop a validated clinical pathway to provide a standardised framework for the transition of care, involving institutional stakeholders.

### 3.2 Provider‐Level Integration: Hospital and Centre Coordination

1. Establish and organise joint multidisciplinary handover meetings between paediatric and adult specialists within the same centre (for centres housing both clinical domains) to ensure intra‐institutional continuity of care.
2. Paediatric specialists should identify a formal network of adult‐care professionals to guarantee an optimal transition and to ensure continuous and qualified care.
3. Promote both awareness and specialised training among adult‐care professionals (specialists and general practitioners [GPs]) regarding RDs, including, whenever possible, a PO perspective, aimed at mitigating the perceived care gap and easing/reducing the distress patients face during transition.
4. Enhance inter‐specialist communication through the implementation of digital tools, including the Patient Health Profile (PHP) and telemedicine platforms.

### 3.3 Clinician‐Led Transition Support

1. Provide gradual guidance for patients and families throughout the transition process, offering psychological support as well as informational meetings and multimodal resources—including dedicated web pages, educational videos and infographics—co‐organised by clinical referral centres and patient advocacy groups.
2. Actively engage patients and caregivers in the transition pathway, ensuring awareness of key steps and their respective responsibilities.

### 3.4 Advocacy‐Driven Patient Empowerment and Peer Support

1. Promote patient awareness and self‐care to encourage autonomy in independent health management and in participation in clinical research activities.
2. Establish a peer‐mentoring framework (intergenerational mentoring) whereby patients who have faced and completed the transition process provide guidance and psychosocial support to those currently undergoing it, sharing experiential knowledge and practical strategies.
3. Implement structured training programmes for caregivers to equip them with the competencies needed to support adolescents during the transition phase, thereby fostering a family‐centred and integrated care approach.

It is important to note, however, that these groups do not imply exclusive responsibility; rather, they represent a model of shared accountability where multiple stakeholders often collaborate across different levels to achieve the goal.

## 4 Discussion

The transition care management from childhood to adulthood has been investigated by several studies in different fields, with a variety of approaches. As clearly previously stated in the literature, the transition phase from child to adult care is a challenging aspect for people dealing with RBDs [15, 16, 17], such as multiple osteochondromas, Ollier disease and Maffucci syndrome. This liminality phase is indeed fraught with numerous issues that can lead to care discontinuation and sometimes a sense of abandonment in patients and their families [7, 18]. Among those challenges, we can mention the lack of communication between healthcare providers, the emergence of new needs and the demand for a multidisciplinary approach [18].

Some studies approached this aspect by listening to patients and caregivers voices [18], while others investigated transition challenges considering HCPs or GPs opinions [8, 15, 19, 20].

As Scognamiglio et al. [12] have explained, ODA resulted in a series of priorities, which have subsequently been addressed through the DPM, with the involvement of HCPs, POs, patients and caregivers. It should be noted that the ODA study, from which these priorities derive, presents inherent limitations, including a small sample size and disease‐specific scope, that restrict its generalisability. Taking this into account, the present work accepts those findings as a validated starting point for co‐production within the same community, rather than aiming to overcome them. The DPM resulted in several operational solutions that have been organised in the results section according to the entity primarily responsible for their implementation. Nevertheless, these solutions constitute measures to address the ODA priorities in the short‐ and medium‐term.

ODA results identify psychological support as the primary necessity during the transition from paediatric to adult care. For patients, this requirement emerges from the emotional burden of increased medical autonomy and heightened awareness of long‐term risks, including malignant transformation. These findings align with the French consensus guidelines [8] for rare pulmonary diseases, which formally establish that psychosocial follow‐up should be offered at least annually. In line with existing literature [21], the operational solutions proposed in our paper centre on active clinician engagement to facilitate a seamless transition. This requires gradual, multimodal guidance—such as co‐organised educational resources—and the direct, structured involvement of both patients and caregivers.

As the transition marks a shift from a family‐centred to an adult‐oriented model, caregivers must be actively supported in gradually decreasing their direct involvement while remaining a crucial source of encouragement for the patient's emerging independence. Concurrently, caregivers require adequate support to adapt to their children's growing self‐management. Recent French consensus guidelines for rare pulmonary diseases emphasise that parents must gradually take a less active role while remaining an essential source of support. However, these clinical frameworks often lack specific operational strategies to facilitate this shift. Our work addresses this gap by explicitly proposing POs as the primary drivers for delivering structured caregiver training. This approach actively expands the widely recognised educational and supportive role of patient groups, positioning them as key actors for advocacy and mentoring. Moreover, peer‐mentoring initiatives and peer support networks can effectively assist patients during this vulnerable phase. This strategy is strongly endorsed by other recent transition guidelines across different rare conditions, which emphasise the pivotal role of connecting young persons with peer support groups to enhance resilience and self‐advocacy [3, 21].

The second ODA priority was the need for a defined, tailored and planned care pathway to accompany patients during the transition process, as also stated by literature [9, 10, 11, 12, 13, 14, 15, 16, 17, 22, 23]. This aspect is more complicated, considering that it requires actions at multiple levels, including the system level representing, by its own nature, represents the most demanding, complex and time‐consuming. The TMC is not a mere transfer of patients from one HCP to another, or from one specialist to another. As stated by Blum in 1993 [24], the transition represents a ‘purposeful, planned process that addresses the medical, psychosocial and educational/vocational needs of adolescents and young adults with chronic physical and medical conditions as they move from child‐centred to adult‐oriented healthcare systems’. As Choukair et al [25] presented for rare endocrine conditions, the absence of care pathways can impact multiple aspects of patients’ lives in the short and long term. These impacts can include poor clinical management, worsening quality of life and additional hospitalisations. Moreover, while the potential costs and investments related to its implementation are present, they are limited and less impactful than the costs associated with the worsening of the disease or medical complications in case of abrupt transfer [21]. To address this ODA priority, the DPM has identified several actions at different levels, helping in channelling adolescents into a planned care pathway. Considering institutions with children and adult specialists, a suitable solution would be to implement a multidisciplinary handover meeting between the relevant specialists. This could be complemented by a joint visit to the outpatient clinic involving both paediatric and adult specialists [25, 26]. For paediatric centres, an operational solution for promoting a fruitful care pathway and guaranteeing a proper transition is the establishment of a formal network of adult‐care specialists. Such a network could provide paediatric specialists with valuable support to help them address the transition properly and reduce—or even eliminate—the sense of abandonment patients may experience during this complex phase of their lives.

Another system‐level solution that has been formulated as a result of the DPM is the production of a PHP—sometimes referred to as a structured epicrisis or transition booklet—that summarises the course of the disease to date, including surgical history, ongoing treatments, molecular results, as well as clinical contact information. The PHP should be accessible to HCPs, patients and caregivers (when appropriate). This practical approach has been suggested with minor differences by several studies [17, 18, 19, 20, 21, 24, 25, 27, 28, 29, 30]. Indeed, given that the PHP can improve communication between specialists, it would be extremely beneficial in the transition phase, as well as in other sensitive situations where patients are transferred between different referral centres for unrelated reasons (e.g., second opinion).

The last solution that arose related to the establishment of a care pathway during the transitioning phase is the promotion of awareness and dedicated training among specialists as well as GPs. Indeed, as reported by Puyraimond‐Zemmour and by Peulier‐Maitre [8, 27], GPs, despite their infrequent involvement, can make a substantial contribution to the provision of continuity of care. GPs, when properly trained and involved in transitioning, are capable of identifying pathway disruptions, facilitating coordination among specialists and settings and offering channels for psychological support.

Whilst none of the aforementioned solutions can wholly supplant the establishment of a structured transition pathway, they can function as preliminary operational steps to provide support and pave the way for establishing a validated care pathway to ensure proper transitioning and adequate continuity of care. These solutions can also support rare patients in a variety of settings that extend beyond the TCM, such as reproductive care or the onset of new clinical manifestations.

From a methodological standpoint, the present study presents several limitations. The panel composition reflects those professionals who responded to the invitation; as the five non‐respondents did not provide reasons for their non‐participation, a degree of selection bias cannot be formally excluded. Furthermore, the analytical process relied on an affinity grouping approach and internal synthesis conducted by ACAR Aps, rather than on a formally validated qualitative method. Future efforts to implement and evaluate these solutions in clinical settings would benefit from the adoption of established implementation frameworks, such as the Consolidated Framework for Implementation Research (CFIR) [31] or RE‐AIM [32], to systematically assess facilitators, barriers and reach across different healthcare contexts.

## 5 Conclusions

Through this DPM, we have established a structured yet flexible environment for collaborative consensus‐building. This approach supports the transition from preliminary qualitative findings toward the implementation of actionable, community‐endorsed, operational solutions for RBD care. Achieving a successful transition from childhood to adulthood is a topic of interest in many European countries. Establishing specific, realistic and achievable goals, which are also accepted and shared by all relevant stakeholders, is a significant step forward in ensuring continuity of care for patients affected by multiple osteochondromas, Ollier disease and Maffucci syndrome, as well as for other rare bone patients.

## Author Contributions

**Marina Mordenti:** writing – original draft, investigation, conceptualisation, methodology, formal analysis. **Silvia Forni:** conceptualisation, investigation, writing – original draft, methodology, project administration, formal analysis. **Eleonora Grippa:** conceptualisation, investigation, writing – review and editing, methodology, project administration, formal analysis. **Maria Cecilia la Forgia:** writing – review and editing, investigation, formal analysis. **Davide Scognamiglio:** investigation, writing – review and editing, formal analysis. **Manila Boarini:** investigation, writing – review and editing, methodology, conceptualisation, formal analysis. **Gabriella Massa:** writing – review and editing, investigation, formal analysis. **Andrea Romeo:** conceptualisation, investigation, writing – review and editing, formal analysis. **Alessandro Sergi:** conceptualisation, writing – review and editing, methodology. **Luca Sangiorgi:** conceptualisation, investigation, funding acquisition, writing – review and editing, methodology, formal analysis. **Giovanni Beltrami:** investigation, writing – review and editing. **Domenico Campanacci:** investigation, writing – review and editing. **Nunzio Catena:** investigation, writing – review and editing. **Mauro Celli:** investigation, writing – review and editing. **Marta Cervo:** investigation, writing – review and editing. **Leonardo D'Agruma:** investigation, writing – review and editing. **Gian Luigi Federico:** investigation, writing – review and editing. **Giovanni Gallone:** investigation, writing – review and editing. **Michela Veronika Gonfiantini:** investigation, writing – review and editing. **Laura Masi:** investigation, writing – review and editing. **Maria Beatrice Michelis:** investigation, writing – review and editing. **Osvaldo Palmacci:** investigation, writing – review and editing. **Annabruna Ronchetti:** investigation, writing – review and editing. **Paolo Trezza:** investigation, writing – review and editing.

## TCM WG Collaborators

Giovanni Beltrami, MD (Università di Firenze, AOU Meyer, Firenze, Italy); Domenico Campanacci, MD (Università di Firenze, AOU Careggi, Firenze, Italy); Nunzio Catena, MD, MSc (IRCCS Istituto Giannina Gaslini, Genova, Italy); Mauro Celli, MD (Policlinico Umberto I, Roma, Italy); Marta Cervo, PT (AOU Meyer, Firenze, Italy); Leonardo D'Agruma, BSc (Fondazione IRCCS ‘Casa Sollievo della Sofferenza’, San Giovanni Rotondo, Italy); Gian Luigi Federico, MD (AORN Santobono, Napoli, Italy); Giovanni Gallone, MD (IRCCS Istituto Ortopedico Rizzoli, Bologna, Italy); Michela Veronika Gonfiantini, MD (Ospedale Pediatrico Bambino Gesù, Roma, Italy); Laura Masi, MD (Università di Firenze, AOU Careggi, Firenze, Italy); Maria Beatrice Michelis, MD (IRCCS Istituto Giannina Gaslini, Genova, Italy); Osvaldo Palmacci, MD (Fondazione Policlinico Universitario A. Gemelli IRCCS, Università Cattolica del Sacro Cuore, Roma, Italy); Annabruna Ronchetti, MD (IRCCS Istituto Giannina Gaslini, Genova, Italy); and Paolo Trezza, MD (Presidio Ospedaliero Gaetano Pini, Milano, Italy).

## Ethics Statement

The authors have nothing to report.

## Consent

A **s**pecific authorisation for the use of images from patients, families and caregivers was collected during the XV ACAR Aps meeting roundtable, April 2025, with the aim of creating a detailed record of the points discussed and supporting a summary of the identified solutions.

## Conflicts of Interest

The authors declare no conflicts of interest.

## Data Availability

Data sharing is not applicable to this article as no datasets were generated or analysed during the current study. Full interview transcripts and videoconference recordings are not publicly available due to data protection regulations.
